# The assessment of the concentration of candidate cytokines in response to conjunctival-exposure of atmospheric low-temperature plasma in an animal model

**DOI:** 10.1186/s12886-021-02167-z

**Published:** 2021-12-04

**Authors:** Farhad Nejat, Khosrow Jadidi, Hosein Aghamollaei, Mohammad Amin Nejat, Nazanin-Sadat Nabavi, Shima Eghtedari

**Affiliations:** Vision Health Research Center, Semnan University of Medical Sciences, Semnan, Iran

**Keywords:** Atmospheric low-temperature plasma, Inflammatory responses, Interleukins, Ocular surface tissues, Conjunctival tissues

## Abstract

**Background:**

Atmospheric Low-Temperature Plasma (ALTP) can be used as an effective tool in conjunctival cyst ablation, but little is known about how conjunctival ALTP-exposure affects the concentration of inflammatory mediators and also the duration of inflammatory responses.

**Methods:**

We used 8 female adult Lewis rats that were followed up in 4 groups. The right eye of each rat was selected for the test, whereas the left eye was considered as a control. The ALTP was generated and used to target 3 spots of the conjunctiva. The digital camera examinations were performed to follow-up the clinical outcomes after ALTP exposure. Tear and serum samples were isolated—at 2 days, 1 week, 1 month, and 6 months after treatment—from each rat and the concentration of candidate pro-inflammatory (i.e. IL-1α, IL-2, IL-6, IFN-γ, and TNF-α) and anti-inflammatory cytokines (i.e. IL-4 and IL-10) were measured using flow cytometry.

**Results:**

The external and digital camera examinations showed no ocular surface complications in all ALTP-exposed rats after 1 week. The analyses revealed that the ALTP transiently increases the concentration of pro-inflammatory cytokines—IL-1α and IL-2 in tear samples in 1 week and 2 days after exposure, respectively; no differences were observed regarding other pro- and anti-inflammatory cytokines in the tear or serum samples.

**Conclusions:**

ALTP can probably be used as a minimally-invasive therapeutic method that triggers no permanent or continual inflammatory responses. The results of this study might help the patients to shorten the consumption of immunosuppressive drugs, e.g. corticosteroids, that are prescribed to mitigate the inflammation after ALTP-surgery.

**Supplementary Information:**

The online version contains supplementary material available at 10.1186/s12886-021-02167-z.

## Introduction

Plasma is the fourth state of matter that consists of roughly equal numbers of positively and negatively charged particles [1, 2]. This matter can be generated typically at very high or low-temperatures and acquired either naturally or artificially. Nowadays, electrical current in physiological temperatures is often used to produce plasma—which is known as non-equilibrium (non-thermal) atmospheric plasma [3]. This type of plasma is in the same properties as the plasma generated at high temperatures [3]. Plasmas have indeed been used in a span of decades for sterilization of medical equipment or sterile packaging in the food industry [4]; however, their potential applications in medicine have recently attracted much attention; for example, plasma can be used for therapeutic purposes in dentistry as a tooth bleaching agent [5], in dental plaque treatment [5], cancer-therapy [6], wound-healing of autoimmune diseases [7], disinfection and blood coagulation [8], and impaired tissue ablation [9].

As a subgroup that uses atmospheric air instead of a noble gas, atmospheric low-temperature plasma (ALTP) produced at atmospheric pressure is supposed to be safe for use on physiological conditions [10]. In fact, LTP application has broadened the horizons toward modern plasma medicine that is frequently used in divergent fields including wound-healing, ophthalmology, otolaryngology, gastroenterology, odontology, dermatology, and even cancer treatment (reviewed in [11–13]). Additionally, Brehmer et al. reported that the LTP treatment was invulnerable and executable in patients with chronic venous leg ulcers [14], substantiating the medical utilization of this novel approach. In general, the effects of the LTP on biological procedures depend on the targeted cell and tissue [15], the composition of plasma used [16], discharge dose, and the shape of the voltage used to the discharge [17].

There is an urgent need for effective therapeutic approaches that can, in turn, modulate eye diseases, e.g. eye infection, while simultaneously mitigating the side-effects including ulceration, extended inflammation, and surrounded tissue necrosis [18]. ALTP can indeed be considered as a novel approach to surgery or to eliminate the implications of the ocular surface tissues that need a procedure with a high success rate [19]. Beyond that, understanding the underlying mechanisms of ALTP-cell interactions is necessary and vital to assure safety during ALTP treatment. In sum, although the effect of LTP on various biological processes has been peered at, its impressions on inflammatory responses and also its potential applications in ophthalmology are still unclear.

It has been demonstrated that the cold atmospheric plasma-treatment can promote the levels of the pro-inflammatory cytokine in the skin wound-healing process, specifically, the levels of pro-inflammatory factors such as tumor necrosis factor-α (TNF-α) and interleukin-1β (IL-1β) [20]. Although in our previous study, we confirmed the safety of the ALTP used for conjunctival cyst ablation [18], there is no information about the effects of ALTP on pro- and/or anti-inflammatory cytokines in the tears or sera of the exposed-animal models. Indeed, this might help us to choose a better drug intervention and minimize the side effects regarding the shortening anti-inflammatory drugs period. To fill this gap, in the present study, we investigated the effects of ALTP exposure on the mucous membrane of the eye—conjunctiva—in an in vivo rat model on the level of pro- and anti-inflammatory cytokines in the tear and serum samples.

## Materials and methods

### Animals

The research project was approved by the ethics committee of Semnan University, Semnan, Iran. This study is also in line with the criteria of the National Institutes of Health guide (NIH Publications No. 8023, revised 1978) for the care and use of laboratory animals [21]. In this study, 8 female adult Lewis rats (90 days old) were used—weighing 200–250 g—that were handled according to the Association for Research in Vision and Ophthalmology (ARVO) [22]. All procedures were also carried out in compliance with the ARRIVE guidelines.

The rats were fed ad libitum and were kept in standard conditions. All surgical procedures were performed under anesthesia, and all efforts were made to minimize the suffering of the animals. Rats were separately housed in isolated cages under standard conditions containing room temperature (25–28 °C), recommended humidity (50–60%), and light-dark period of 12 h.

### Experiments

Before embarking on the experiment, all rats were meticulously evaluated for eye complications and anterior chamber inflammation. Prior to exposing the ALTP, we used intraperitoneal injections of 40 mg/kg ketamine hydrochloride (Ketaset, 100 mg/ml; Pfizer, NY, USA) and 6–8 mg/kg xylazine (Chanazine, 20 mg/ml; LLOYD Laboratories, IL, USA) to anesthetize the rats. This ketamine-xylazine mix was optimally vortexed and prepared fresh on the day of procedure and was injected to the candidate rats. The rats were continuously observed throughout the procedure of anesthesia by monitoring the body temperature, heart rate, and respiratory rate.

The right eye of each rat was selected for the test, while the left one was considered as the control. Experiments were carried out using the Plexr device (GMV, Rocca Priora, RM, Italy) by targeting 3 spots of the conjunctiva at 11–13 o’clock positions (Supplementary Video 1). The Plexr device was in the continuous mode and also in the lowest power level (White handpiece; Vpp = 500 V, Power = 0.7 W, and Frequency = 75 kHz); it was used at 0.7 s intervals using a 22-gauge needle; all detailed information is put forward in Table 1. The Plexr used the sublimation process whereby the solid phase (without any transitional liquid phases) is directly converted to gas, hence impeding any thermal damage to the surrounding tissue. This sublimation occurs when the air between the tip of the device and target tissue become ionized.

**Table 1:** Technical features of the Plexr device that was used in this study

Characteristics	Values
Working gas	Air
Power supply	Docking station = 24 V
	Handpieces: embedded inductive charger = 5 V
Handpieces	
Max output	≤ 2 W
Max working voltage	≤ 1.3 k VPP
Output frequency	(70–80) kHz
Handpiece types	
White^a^	V peak to peak = 500 V, Power = 0.7 W, Frequency = 75 kHz
Green	V peak to peak = 600 V, Power = 1 W, Frequency = 75 kHz
Red	V peak to peak = 700 V, Power = 2 W, Frequency = 75 kHz
Maximum absorbed power (Docking station)	120 W
Applicator electrode	Stainless steel sterile disposable needle
Risk classification of the device	medium-to-high risk

^a^in the present study, we used the ‘White’ handpiece


**Additional file 1: Supplementary video 1.** The procedure in which the right eye of the rats was exposed using the Plexr device. The conjunctiva was chosen to be exposed by ALTP.

Chloramphenicol as eye drops was used to treat rats every 6 h for 1 week so as to prevent any eye infections. To detect any abnormality, they were checked for abnormal behavior every day. At the end of the experiment, the rats were sacrificed by intraperitoneal injection of a lethal dose of pentobarbital (Tocris Cookson Ltd., Bristol, UK).

### External examinations

To scrutinize the effects of ALTP on ocular surfaces, the eyes were evaluated for any symptoms of conjunctival chemosis, redness, discharge, and also lid-swelling after 2 days (2D; group A), 1 week (1 W; group B), 1 month (1 M; group C), and 6 months (6 M; Group D) exposure to plasma. Since the ALTP effects are supposed to be limited to the initial days after exposure, to be on the safe side, the clinical evaluations after 24 and 48 exposure were performed. Draize scoring was also used to evaluate the level of injuries to conjunctiva [23]. In detail, the scores of the observed ocular irritation range from 0 to 3 for conjunctival effects including redness and vessel discernibility [23, 24].

### Digital camera examinations

Selected eyes were photographed using a digital camera so that we identify the surface abnormalities like infiltration and inflammation and also to follow-up the wound-healing process of the conjunctiva. A cobalt blue filter was used at 3 min after using a standard fluorescein strip (Jingming, China) on the ocular surface, as a result, the plasma-generated epithelial defects were elucidated using the areas with positive staining. In this step, all evaluations were meticulously performed by an ophthalmologist.

### Tear and serum isolation

Tear samples were collected from each rat using 2 × 5 × 2 mm sterile sponges (Weck cel, NY, USA) that were placed between the eyeball and the lower conjunctiva for 30–60 s. Before sampling, around 50 μl of the sterile physiological serum was used for washing the eyes. Subsequently, 50 μl of sterile PBS was directly dropped into each eye, and the tear fluid was extracted using sterile sponges followed by centrifuging at 10,000×g for 10 min. A sample was taken from each right and left eye at the aforementioned periods after ALTP exposure. The tear samples were pooled and stored at − 80 °C until further investigations.

Beyond that, blood samples were taken using a syringe with a small needle (with the length of 3/16-in.) from each rat and were centrifuged at 3000×g for 10 min. The serum was isolated and stored at − 80 °C for further investigations.

### Cytometric beads Array

The candidate cytokines including pro-inflammatory (i.e. IL-1, IL-2, IL-6, IFN-γ, TNF-α) and anti-inflammatory cytokines (i.e. IL-4 and IL-10) were evaluated using the BD Cytometric Bead Array (BD Bioscience, San Jose, California, USA). The concentration of each cytokine was calculated according to pg/ml using the standard curves. Briefly, 100 μl tear and serum fluid was thawed and transferred to a 50 μl of each capture antibody-bead reagent. We added 50 μl of sterile PBS into the tear samples by the way. The mixture subsequently was incubated for 1 h at room temperature. Then, the antibody-phycoerythrin (PE) reagent was added to each sample and incubated for 2 h at room temperature followed by washing to cut down on the unbound antibodies. Flow cytometric analyses were performed using a FACSCalibur® flow cytometer (Becton Dickinson Immunocytometry Systems, San Jose, CA) according to the previous studies ^26^. The following antibodies were used: interleukin-1α (IL-1α; Abcam Biotech Co. Ltd., Cambridge, MA, USA), IL-2 (R&D Systems Biotech Co. Ltd., Emeryville, USA), IL-4 (R&D Systems Biotech Co. Ltd., Emeryville, USA), IL-6 (R&D Systems Biotech Co. Ltd., Emeryville, USA), IL-10 (Abcam Biotech Co. Ltd., Cambridge, MA, USA), TNF-α (R&D Systems Biotech Co. Ltd., Emeryville, USA), and IFN-γ (R&D Systems Biotech Co. Ltd., Emeryville, USA). The lower amount of the detection range were as following: IL-1α, 2.6 pg/ml; IL-2, 2.1 pg/ml; IL-6, 2.04 pg/ml; IFN-γ, 1.2 pg/ml; TNF-α, 1.6 pg/ml; IL-4, 1.5 pg/ml; and IL-10, 2.5 pg/ml. Data were acquired and analyzed for obtaining the cytokine concentration based on the standard curves.

### Statistical analysis

All results were reported as means ± Standard Division (SD) and analyzed using the SPSS software Version 24.0 (IBM, Armonk, NY, USA). Mann–Whitney test was used and *P*-values ≤0.05 were considered statistically significant. All graphs were depicted employing the GraphPad Prism software package (GraphPad Software Inc., San Diego, CA, US).

## Results

### External eye-examination

**Fig. 1:**
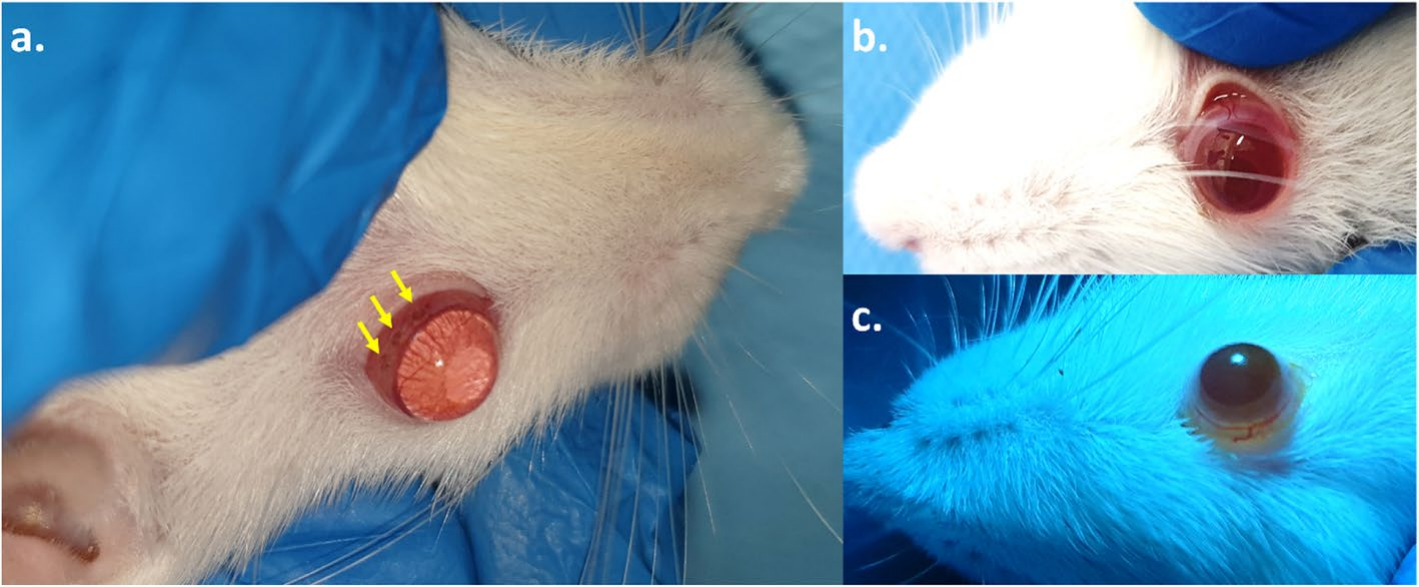
The ALTP was used to target the conjunctiva. **a** The right eye of each rat was selected for the test by targeting 3 spots of the conjunctiva at 11–13 o’clock positions (yellow arrows). **b** the left eye was used as the control. **c** the putative spots were not observable after 1 week. No abnormal irritations/complications including conjunctival chemosis, redness, discharge, and lid swelling were also identified

The ocular surface did not show any complications in all groups of rats for conjunctival chemosis and discharge (Score 0). As a transient symptom, the redness was observed on the first day (Score 1) (Fig. 1). Besides, mild eyelid swelling was also observable in all treated rats. After exposing ALTP and during the first 3 days, the rats suffered from photophobia, but in the following days, the light sensitivity vanished.

### Digital camera examinations

Doing fluorescein staining, the conjunctival epithelial defect (~ 0.8 mm) was detectable owing to the ALTP-exposure. Neither infiltration nor extended observable inflammation was detected during the study. We also examined the anterior chamber reaction, although it was negative for all groups. Although the spots were evident at 48 h after exposure, they were not observable after 1 week. Further following up— at 1 month and 6 months after ALTP-exposure— did not show any complications.

### The concentration of cytokines in tear and serum samples

**Fig. 2:**
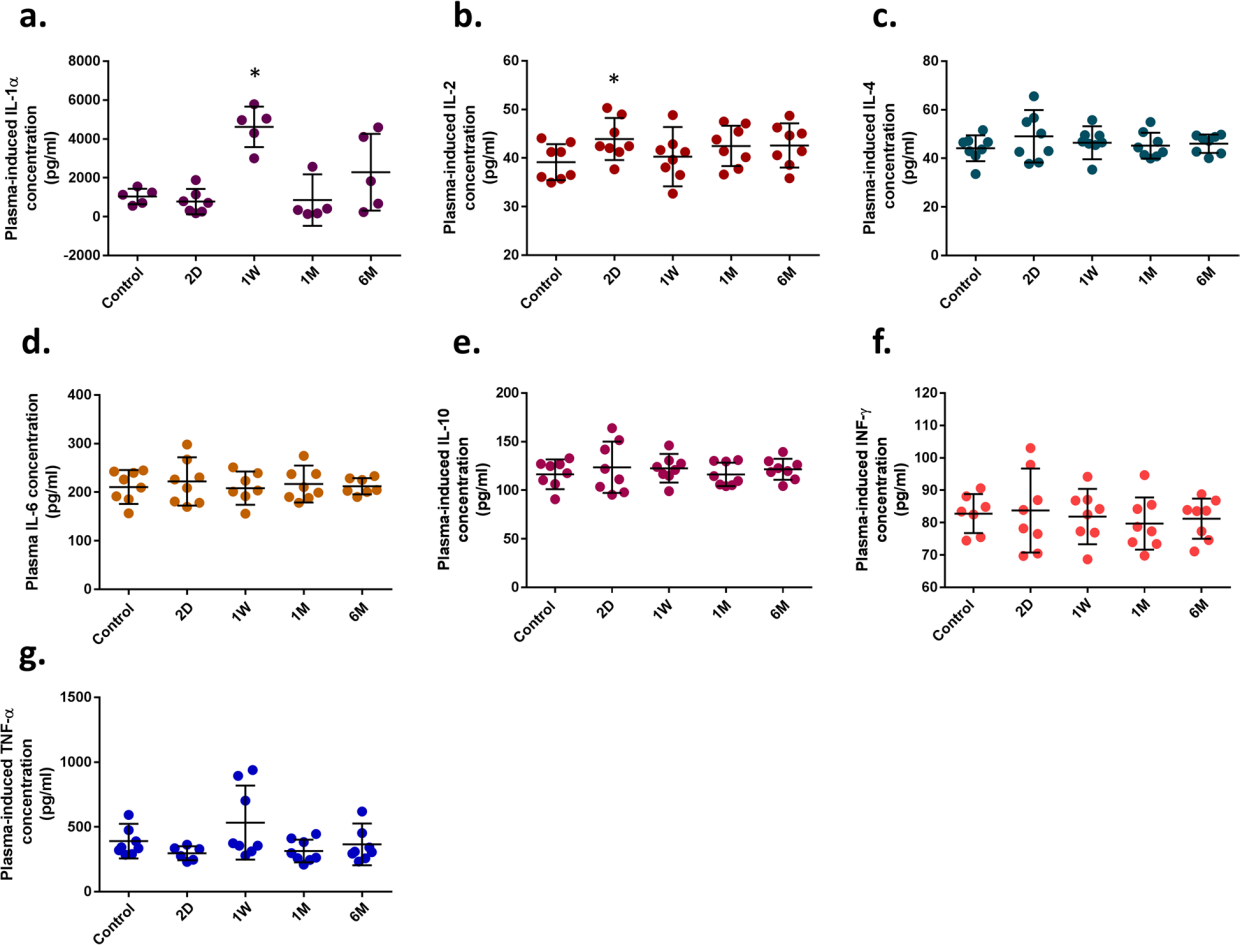
Flow cytometry was used to measure the candidate cytokine concentration in tear samples. **A** there is a significant increase in tear IL-1α concentration (*P*-value < 0.05) at 1 week (1 W; group **B**) after ALTP exposure. **B** The concentration of IL-2 was increased at 2 days (2D; group **A**) after ALTP-exposure. Comparison between the candidate and control groups showed no significant differences in IL-4 (**C**), IL-6 (**D**), IL-10 (**E**), INF-γ (**F**), and TNF-α (**G**). In this figure: an asterisk (*) indicates *P*-values < 0.05, 2D: 2 days after ALTP-exposure, 1 W: 7 days after ALTP-exposure, 1 M: 1 month after ALTP-exposure, and 6 M: 6 months after ALTP-exposure. The data represent the mean ± SD

**Fig. 3:**
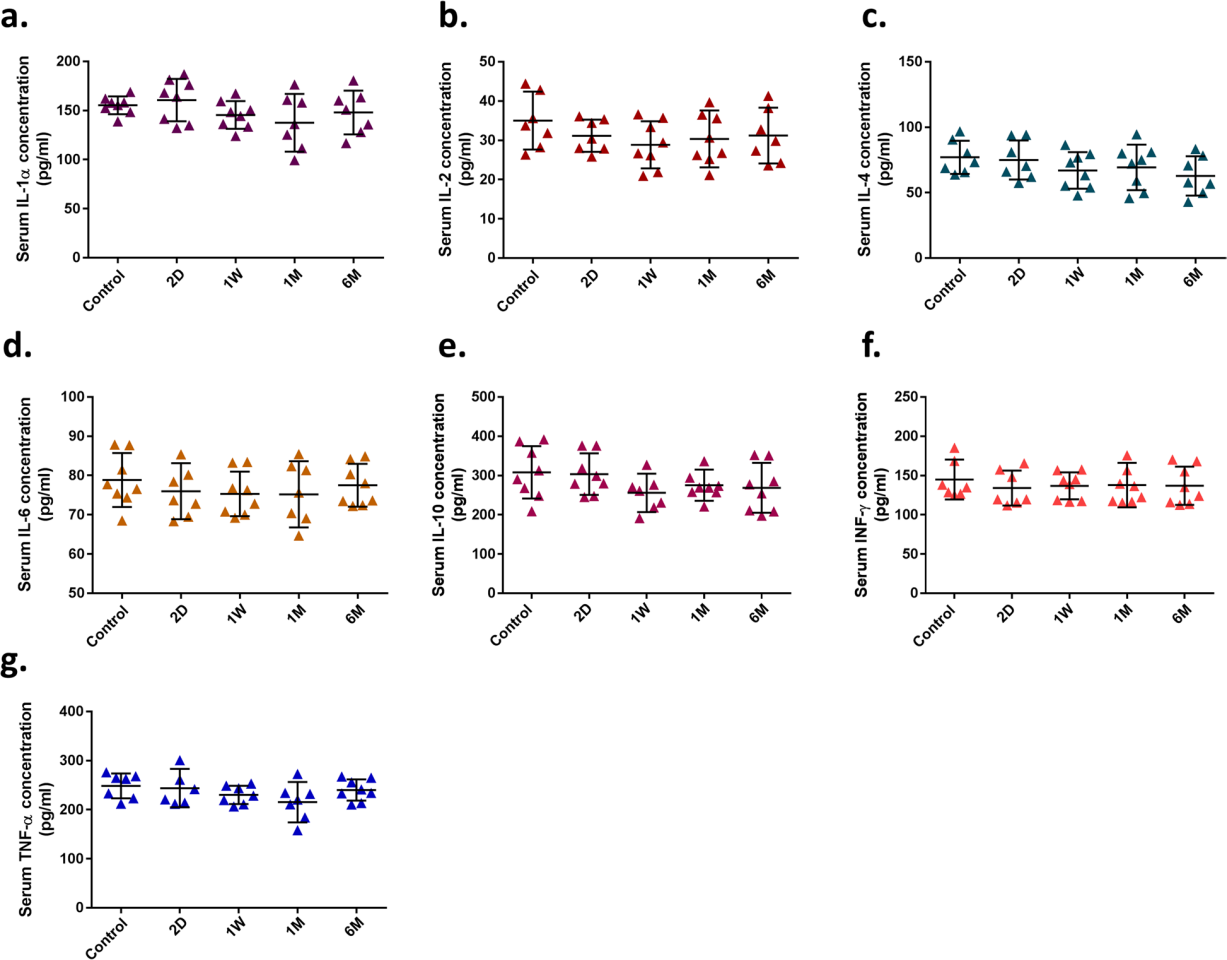
The concentration of candidate cytokines in serum samples were measured by flow cytometry. The histograms show the average serum cytokine concentration of 8 rats. No significant differences were observed in IL-1α (**A**), IL-2 (**B**), IL-4 (**C**), IL-6 (**D**), IL-10 (**E**), INF-γ (**F**), and TNF-α (**G**). The data represent the mean ± SD

All seven cytokines were detectable in tear samples derived from the 8 rats; their concentrations, according to pg/ml, are put forth in Fig. 2a-g. Tear samples derived from group B (1 W) had significantly higher IL-1α concentration (4621.2 pg/ml in average) in comparison with the control group (1039.3 pg/ml in average) (*P*-value < 0.05; Fold Change_1W/control_ = 4.44). Furthermore, we indicated that the concentration of IL-2 (43.90 pg/ml in average) increased in group A (2D; *P*-value < 0.03; Fold Change_2D/control_ = 1.12), while in other groups, no differences were detected (in average, the concentration of IL-2 was 39.14 pg/ml in control, 40.29 pg/ml in 1 W, 41.35 pg/ml in 1 M, and 41.46 pg/ml in 6 M groups). No significant differences in concentrations of IL-4, IL-6, IL-10, TNF-α, and IFN-γ cytokines were found in tear samples (Fig. 2b-g). All cytokines were in detectable range in serum samples, while no significant difference was detected among all candidate groups (Fig. 3a-g).

## Discussion

To treat ocular surface diseases, various interventions (e.g. surgery) have been performed that, to some extent, are the potential to increase the disease deterioration [25]. In recent years, it has been repeatedly demonstrated that LTP exposed on the surface of the eye may eliminate microorganisms without any destructive effects on the external eye tissues [26–29]. Revolutionary, this has extended the expectations to use the LTP or even cold atmospheric plasma in order to treat ocular surface diseases. For instance, some scrapes of evidence bear out when argon cold atmospheric plasma was employed on the ocular surface, it causes no impaired corneal epithelial cells in vitro, ex vivo, and also in patients’ eyes [30]. Molecularly, it has been elucidated that using the low temperature or even cold atmospheric plasma can induce transient expression of some genes responsible for oxidative stress [31], promote localized/limited apoptotic responses [32], and trigger a minimal decrease of cell viability [33], all are negligible in comparison with the conventional methods to treat ocular surface diseases.

A shred of accumulating evidence suggests that inflammation takes a center stage in the clinically exacerbation of patients who underwent the surgery for ocular surface diseases [34]. Ocular surface cells themselves express and produce inflammatory mediators such as cytokines, e.g. IL-1α, IL-2, and TNF-α [35]. In various eye complications such as alkali burn [36] and herpetic stromal keratitis [37], the concentration of different inflammatory cytokines, e.g. IL-1α and IL-6, are dramatically elevated, showing that inflammation is a sine qua non of eye complications and/or eye surgery; to eliminate, anti-inflammatory therapy is the first line of treatment. Arndt et al. showed that LTP exposure can increase the gene expression of regulators that are important for inflammation and wound-healing without causing proliferation, migration, or cell death in keratinocytes [38]. Using Human Osteosarcoma Cells, it has also been found that 15 inflammatory cytokines, particularly embracing IL-1α, increased when the cells were treated with cold atmospheric plasma [39]. These studies raise a point of whether LTP- or even cold atmospheric plasma-exposure can modulate inflammatory responses or not.

The concentration of inflammatory cytokines was measured in the tear and serum samples afterward. The results of flow-cytometry showed that IL-1α increased in group B (1 W) in tear samples but not in the other groups, i.e. the concentration of IL-1α came back to the normal levels (Fig. 2a); it confirms that ALTP cannot permanently or even for a long time promote the secretion/expression of IL-1α in tear samples. On the other hand, no significant differences were observed in serum samples; it can be suggested that the ALTP affects inflammatory responses around the operated site/organ, e.g. eye and tear here. According to Lam et al.*,* the severity of the ocular surface epithelial disease is related to various cytokines and chemokines, including IFN-γ, IL-1α, IL-1β, and IL-6. These cytokines may signal ocular surface stress and can affect neural sensitivity and cause hyperalgesia [40]. We could not find any significant changes of IL-1α in sera; this can also demonstrate that ALTP exposure transiently increases IL-1α concentration in the tear or near the ALTP-exposed regions.

Similarly, as a pro-inflammatory cytokine, IL-2 just increased in 2D groups in tear samples. IL-2 was identified as a cytokine that had multiple effects in immunomodulation, e.g. it can stimulate natural killer (NK) cells and B cell growth factor activity which might stimulate B cell activation [41]. IL-2 probably contributes to the ocular immune responses, peculiarly in the presence of activated T cells in the uvea and peripheral blood of patients with active uveitis [42] and its crucial role in T cell-mediated immunity [43]. IL-2 has innate effects such as vascular leakage and the stimulation of neutrophils and macrophages, in addition to immune-stimulating effects such as antigen presentation and T cell proliferation [44]. Immunosuppressive drugs, e.g. corticosteroids, that are prescribed after ocular surgery often inhibit the synthesis of IL-2 and IFN-γ [45, 46]; for instance, Daclizumab has proved its efficacy in mitigating IL-2 concentration in patients with ocular complications [47]. Consuming such immunosuppressive drugs by patients who underwent ocular surgery for a long time can increase the infection the risk of infection, gastrointestinal and renal upset, bone marrow suppression, hypertension, hematuria, infusion/hypersensitivity reactions, and so on [48]. Under certain assumptions, corticosteroids can also induce glaucoma and cataract formation, and delay ocular wound healing [49]. Our study suggests that increased inflammatory responses are detectable in tear samples no longer than 7 days after ALTP-exposure, this might be construed as prescribing or taking immunosuppressive drugs in patients in this time range to minimize the side effects as much as possible.

In the previous study, we used ALTP to remove the human conjunctival cyst [19]. We showed that using ALTP is advantageous because it is performable under topical anesthesia, does not require the incision and suture, and also is handy and minimally invasive. Moreover, ALTP is a simple, office-based method with a cost-affordable nature that can frequently be used. ALTP application did not cause observable inflammation and necrosis in the scleral and other deep and surrounded tissues [19]. Besides, we previously showed that using ALTP could not trigger the clinically observable inflammation in rabbit eyes [18]. In the present study, we aimed to show whether the concentration of inflammatory mediators—including pro- and anti-inflammatory cytokines—can be affected by the ALTP-exposure or not. Given half a chance to be influenced, how much time would need to return to the normal concentration? To answer, at the first stage, we exposed the ALTP on the conjunctiva via the lowest power level of the Plexr device (white handpiece). Accordingly, in all groups, no serious complications—e.g. conjunctival chemosis and discharge—were detected, substantiating the safeness of the ALTP procedure to treat the ocular surface disease. Herein, the duration of plasma treatment was < 3 s (including 3 spots with a period of about 1 s) and it is unlikely to lead to serious damages.

Regarding other pro-inflammatory (i.e. IL-6, IFN-γ, and TNF-α) and anti-inflammatory cytokines (i.e. IL4 and IL10), we could not find any alternation in their concentration, suggesting that ALTP can effectively be used as a promising method to treat the ocular surface diseases. Even though future studies must clarify how ALTP exactly can change the expression of pro-inflammatory in tear samples.

One of the major drawbacks of our study is the low number of animal models. Further studies are needed to replicate the data using a higher number of animal models. Besides, extending the research to human cases will definitely help us to remove the veil of ignorance. Therefore, studies with more patients and possible longer follow-up time are vital to draw a conclusive result. Another limitation is the limited number of candidate cytokines. We focused on the important candidate pro- and anti-inflammatory cytokines, but future investigations should determine the throughout immune-profile including all important cytokines, chemokines, immune cells, and other inflammatory mediators after ALTP treatment. Last but not least, we spotted three limited regions in conjectiva of rats, while in counterpart study in human or even using rabbit more surface of conjectiva will be available; we believe that future studies can help us to better replicate and follow up the results using animal models or human cases.

## Conclusions

This study provides evidence that a short-time application of ALTP can increase the transient expression of pro-inflammatory cytokine IL-1α and IL-2, no longer than 7 days after ALTP-exposure. Furthermore, no alternation was found of the candidate cytokines in tear or serum samples, suggesting that the inflammatory responses are restricted to the regions in which ALTP was exposed. Taken together, our findings suggest that ALTP with appropriate composition and concentration of plasma agents may be used in clinical trials with trivial inflammatory responses. By doing clinical trials, it may possible to prescribe the anti-inflammatory drugs in a short time to better manage the clinical outcomes; however, further fundamental studies are imperative to answer the questions and to introduce this novel method into clinical practice.

## Data Availability

The datasets used and/or analysed during the current study available from the corresponding author on reasonable request.

## Abbreviations

ALTP: Atmospheric Low-Temperature Plasma
TNF-α: tumor necrosis factor-α
IL-1β: interleukin-1β
